# IGFBP-4 regulates adult skeletal growth in a sex-specific manner

**DOI:** 10.1530/JOE-16-0673

**Published:** 2017-02-09

**Authors:** David E Maridas, Victoria E DeMambro, Phuong T Le, Kenichi Nagano, Roland Baron, Subburaman Mohan, Clifford J Rosen

**Affiliations:** 1 Maine Medical Center Research InstituteScarborough, Maine, USA; 2 Harvard School of Dental MedicineBoston, Massachusetts, USA; 3 VA Loma Linda Healthcare SystemLoma Linda, California, USA

**Keywords:** IGF, IGFBP4, bone, phenotype, mice, gender

## Abstract

Insulin-like growth factor-1 (IGF-1) and its binding proteins are critical mediators of skeletal growth. Insulin-like growth factor-binding protein 4 (IGFBP-4) is highly expressed in osteoblasts and inhibits IGF-1 actions *in vitro*. Yet, *in vivo* studies suggest that it could potentiate IGF-1 and IGF-2 actions. In this study, we hypothesized that IGFBP-4 might potentiate the actions of IGF-1 on the skeleton. To test this, we comprehensively studied 8- and 16-week-old *Igfbp4^−/−^* mice. Both male and female adult *Igfbp4^−/−^* mice had marked growth retardation with reductions in body weight, body and femur lengths, fat proportion and lean mass at 8 and 16 weeks. Marked reductions in aBMD and aBMC were observed in 16-week-old *Igfbp4^−/−^* females, but not in males. Femoral trabecular BV/TV and thickness, cortical fraction and thickness in 16-week-old *Igfbp4^−/−^* females were significantly reduced. However, surprisingly, males had significantly more trabeculae with higher connectivity density than controls. Concordantly, histomorphometry revealed higher bone resorption and lower bone formation in *Igfbp4^−/−^* females. In contrast, *Igfbp4^−/−^* males had lower mineralized surface/bone surface. Femoral expression of *Sost* and circulating levels of sclerostin were reduced but only in *Igfbp4^−/−^* males. Bone marrow stromal cultures from mutants showed increased osteogenesis, whereas osteoclastogenesis was markedly increased in cells from *Igfbp4^−/−^* females but decreased in males. In sum, our results indicate that loss of *Igfbp4* affects mesenchymal stromal cell differentiation, regulates osteoclastogenesis and influences both skeletal development and adult bone maintenance. Thus, IGFBP-4 modulates the skeleton in a gender-specific manner, acting as both a cell autonomous and cell non-autonomous factor.

## Introduction

IGF signaling is a crucial pathway controlling muscle, fat and bone growth and is regulated by 6 binding proteins, IGFBP1–6. The IGF-binding proteins can enhance or inhibit IGF action locally (Gustafsson *et al*. 1999, Tahimic *et al*. 2013). IGFBP-4, which is highly expressed in adipocytes and osteoblasts, is the smallest IGF-binding protein and contains a cleavage site for pregnancy-associated plasma protein A (PAPPA). It also circulates in relatively substantial amounts in both mice and humans. IGFBP-4 can bind IGF-1 and IGF-2 with higher affinity than the IGF receptor but when cleaved by PAPPA, it can no longer sequester IGF (Mohan *et al*. 1995, Zhou *et al*. 2003, Mazerbourg *et al*. 2004).

IGFBP-4 has been reported to be an inhibitor of IGF signaling *in vitro* in several cell culture models (Culouscou & Shoyab 1991, Schiltz *et al*. 1993, Gustafsson *et al*. 1999, Byun *et al*. 2001). However, *in vivo* studies of IGFBP-4 have shown conflicting results. Indeed, systemic injections of IGFBP-4 in mice induced bone formation, whereas local injections inhibited IGF actions on the bone (Miyakoshi *et al*. 2001). Furthermore, overexpression of IGFBP-4 in bone resulted in a decrease in bone formation (Zhang *et al*. 2003). Yet, when *Igfbp4* was knocked out, mice were born with a decreased body weight persisting up to 14 weeks after birth. It was therefore suggested that IGFBP-4 could regulate prenatal growth by stabilizing IGF-2 before being cleaved by PAPPA leading to increased IGF-2 availability (Ning *et al*. 2008). However, the impact of IGFBP-4 depletion on adults remains unclear. Additionally, past studies have not clarified whether deletion of *Igfbp4* affected females and males as well as specific tissues differentially. Because IGF-1 and IGF-2 play different roles during embryonic life and adulthood, IGFBP4 mechanisms may differ in fetal vs adult animals.

To shed light on the role of IGFBP-4 during postnatal development and in adult mice, we investigated the phenotypes of *Igfbp4*-null (*Igfbp4*^−/−^) females and male mice at 8 and 16 weeks of age; when peak bone mass acquisition is known to occur (Klein *et al*. 1998). Our results suggest that the growth retardation caused by loss of *Igfbp4* persists in adult female and male mice. However, the skeletal phenotype of *Igfbp4*-null mice was gender specific and point to a potential role for this binding protein in skeletal growth and maintenance that is both cell autonomous and cell non-autonomous.

## Material and methods

### Animals

*Igfpb4*^−/−^ mice were a gift from Dr Peter Brooks at Maine Medical Center Research Institute and were generated from the KOMP allele project at University of California Davis. They were produced on the C57BL/6J background by deleting exon 2 of *Igfbp4*, which results in the global loss of IGFBP-4. Genotyping strategy consisted in PCR reactions using primer sets for the wild-type allele (forward: GGTAAACTGGCTCGGATTAGGG; reverse: TTGACTGTAGCGGCTGATGTTG) or the null allele (forward: TTGACTGTAGCGGCTGATGTTG; reverse: TGCCACAGTACCTCCCTTCTC) to distinguish between *Igfbp4*^−/−^-null mice, *Igfbp4^+/+^* control littermates or *Igfbp4^+/^*^−^ heterozygous mice. The mice were bred and maintained in a colony under 14:10 h light:darkness cycles at Maine Medical Center Research Institute in polycarbonate cages with sterilized paper bedding. Mice had *ad libitum* access to sterilized water and Teklad Global 18% Protein Rodent Diet (Harlan Laboratories, Indianapolis, IN, USA). Phenotyping of the mice was performed at 8 and 16 weeks of age (*n* = 10). For the ovariectomy study, 8-week-old mice were subjected to ovariectomy or sham operation (*n* = 5–8/surgery/genotype). They were then placed on a standard AIN93M diet (Research Diets, Inc, New Brunswick, NJ, USA), which does not contain phytoestrogens. At 16 weeks of age, mice were killed for harvest. All experimental procedures were approved by the Institutional Animal Care and Use Committee at the Maine Medical Center Research Institute.

### Dual-energy X-ray absorptiometry (DXA)

Dual-energy X-ray absorptiometry (DXA) was performed on 8- and 16-week-old animals to assess the body composition by measuring whole-body areal bone mineral density (aBMD), areal bone mineral content (aBMC), lean mass and fat proportion. Measurements were taken with exclusion of the head using the PIXImus densitometer (GE-Lunar, Fairfield, CT, USA). A phantom standard provided by the manufacturer was used for instrument calibration before each measurement.

### Micro-computed tomography

Microarchitecture of distal trabecular bone and midshaft cortical bone was analyzed in femora from 16-week-old mice by high-resolution micro-computed tomography (μCT) (VivaCT-40, 10-μm resolution; Scanco Medical AG, Bassersdorf, Switzerland). The machine was calibrated weekly using a phantom provided by the manufacturer. Femurs were scanned at an energy level of 55 kVp and intensity of 145 μA, as described previously (Motyl *et al*. 2013). All scans were analyzed using manufacturer software (Scanco, version 4.05) in accordance with published guidelines. Scans for the cortical region were measured at the mid-point of each femur, with an isotropic pixel size of 21 µm and slice thickness of 21 μm and used to calculate the average total cross-sectional area (mm^2^), bone area (mm^2^) and marrow area (mm^2^). For midshaft analysis, the cortical shell was contoured by user-defined threshold and iterated across the 50 slices. Trabecular bone volume fraction and microarchitecture were evaluated in the secondary spongiosa, starting proximately at 0.6 mm proximal to the growth plate and extending proximally 1.5 mm. Approximately 230 consecutive slices were made at 10.5 μm interval at the distal end of the growth plate and extending in a proximal direction, and 180 contiguous slices were selected for analysis.

### Serum proteins

Blood was collected from 16-week-old animals and spun at 1000 ***g*** for 10 min at room temperature. The serum phase was collected and stored at −80°C before being used in assays. IGFBP circulating levels were measured in sera using MILLIPLEX MAP Mouse IGF-Binding Protein Magnetic Bead Panel (EMD Millipore) according to the manufacturer guidelines. Samples were diluted 25-fold before being processed using magnetic beads labeled with antibodies against IGFBP-1, IGFBP-2, IGFBP-3, IGFBP-5 and IGFBP-6. Samples were read using the Luminex 200 System (EMD Millipore) previously calibrated with xPONENT 3.1 compatible Calibration Kit, and performance was verified with the Performance Verification Kit provided by the manufacturer. Total IGF-1 (free and bound), sclerostin and RANKL levels were measured using ELISA kits (IGF-1: Immunodiagnostic Systems, Gaithersburg, MD, USA; Sclerostin: Alpco, Salem, NH, USA, RANKL: R&D Systems) according to the manufacturers’ instructions. Assay sensitivity was 2.8 ng/mL, 7.4 pg/mL and 5 pg/mL for the IGF1, sclerostin and RANKL ELISAs, respectively. The intra-assay variation coefficients were 5.8, 6.4 and 4.3% for the IGF-1, sclerostin and RANKL ELISAs, respectively. The inter-assay variation coefficients were 7.8, 10.25 and 6.8% for the IGF-1, sclerostin and RANKL ELISAs, respectively. All measurements for ELISA were performed in duplicates.

### Histomorphometry

Calcein and demeclocycline were injected intraperitoneally at 20mg/kg of body weight at respectively 9 days and 2 days prior to killing the mice at 16 weeks of age. Tibiae were fixed for 48 h in 10% buffered formalin and stored in 70% ethanol. The fixed tibiae were dehydrated with graded ethanol and acetone before being embedded in methyl methacrylate. 4 μm consecutive sections were stained with von Kossa, 2% toluidine blue, pH 3.7 or left unstained to, respectively, observe mineralization, count osteoblasts, osteoid, osteoclasts and adipocytes or fluorescence labeling. Analysis was performed 200 μm away from the growth plate using the OsteoMeasure analyzing software (Osteometrics Inc, Decatur, GA, USA). Standard nomenclature was used for the report.

### Quantitative PCR

Total RNA was isolated from cell cultures or flash frozen femurs of 16-week-old mice using TRIzol (Life Technologies) method for tissues. Bone marrow contents were not flushed out from femurs prior to RNA isolation. RNA concentrations were estimated using NanoDrop2000 (Thermo Fisher Scientific), and 500 ng RNA was used as template for the reverse transcription reaction into cDNA using High-Capacity cDNA Reverse Transcription Kit (Thermo Fisher Scientific). The cDNA was diluted 1:20 in nanopure water before being used for quantitative PCR. The iQ SYBR Green Supermix and iQ5 thermal cycler detection system (BioRad) were used to quantify RNA expression. GeNorm kit was used to find the most stable housekeeping gene for bone, which was determined as *Hprt*. Primers for the housekeeping gene and the genes of interest were purchased from PrimerDesign (Southampton, UK) and designed to be 95–100% efficient. Sequences for primers were as follows: *Bglap*: sense: ACCTCACAGATGCCAAGCC, antisense: ATCTGGGCTGGGGACTGAG; *Hprt*: sense: TCCTCCTCAGACCGCTTTT, antisense: AGGTATACAAAACAAATCTAGGTCAT; *Igf1r*: sense: TAATGACAACACTTAATAGCAACAG, antisense: GTAAAGCAGAAAAGAGAGAAAGGA; *Igfbp4*: sense: TACCCACGAAGACCTCTTCATC, antisense: GTCTTCCGATCCACACACCA; *Runx2*: sense: ACCTAACAGTCTTCACAAATCCT, antisense: GAGGCGATCAGAGAACAAACTA; *Sost*: sense: TGAGAACAACCAGACCATGAAC, antisense: TCAGGAAGCGGGTGTAGTG; *Sp7*: sense: ATGGCGTCCTCTCTGCTTG, antisense: TTCCCCAGGGTTGTTGAGTC, *Tnfrsf11b*: sense: AAATTGGCTGAGTGTTTTGGTG, antisense: CTGTGTCTCCGTTTTATCCTCTC; *Tnfsf11*: sense: TTTGCACACCTCACCATCAAT, antisense: CCCTTAGTTTTCCGTTGCTTAAC.

### Primary cell cultures

Bone marrow stromal cells (BMSCs) and hematopoietic stem cells (HSCs) were isolated from femurs and tibias from 8-week-old mice. After the bones were harvested and cut at their distal end, bone marrow contents were spun out by centrifuging at 13,000 ***g*** for 1 min. Cell pellets were resuspended in culture media composed of MEMα with 10% FBS and 1% penicillin/streptomycin.

For BMSCs differentiation into osteoblasts, cells were plated in 6-well plates at 1,000,000 cells/cm^2^. Culture media was replenished every other day. Once cultures were confluent, cells were switched to an osteoblast differentiation media consisting of culture media supplemented with 8 mM β-glycerol phosphate (Sigma-Aldrich) and 0.2 mM ascorbic acid (Sigma-Aldrich). After 7 days of differentiation, cells were fixed with 4% PFA in PBS for 12 min at room temperature. Alkaline phosphatase staining was performed using the Leukocyte Alkaline Phosphatase kit (Sigma-Aldrich) according the manufacturer’s guidelines. For von Kossa staining, 5% silver nitrate solution in water was added to the fixed cells for 1 h with a direct light exposure. Cells were washed with water before being placed in a 5% sodium thiosulfate solution in water for 3 min. Cells were washed and mineral deposits were stained brown.

For HSCs differentiation into osteoclasts, cells were plated at 250,000 cells/cm^2^ in 96-well plates (Corning) in culture media supplemented with RANKL and mCSF. Culture media was changed every 72 h. TRAP staining was performed to stain osteoclasts. Briefly, cells were fixed with 2.5% glutaraldehyde solution in PBS for 30 min and washed twice with PBS. The acid phosphatase, leukocytes (TRAP) kit (Sigma-Aldrich) was used for TRAP staining according to the manufacturer’s guidelines. Cells were counted as osteoclasts when positive for TRAP (purple) and with 3 or more nuclei. To assess osteoclastic resorption, cells were plated on the Osteo Assay Surface (Corning) made with an inorganic crystalline calcium phosphate matrix. Surfaces were colored by von Kossa staining, and pictures of wells were taken to observe how much matrix had been resorbed. *In vitro* assays of osteoblastogenesis and osteoclastogenesis were repeated 3 times.

### Statistical analysis

All data are expressed as the mean ± standard error of the mean (s.e.m.). Results were analyzed for statistically significant differences using a Student *t*-test or a two-way ANOVA followed by Bonferroni’s multiple comparison *post hoc* test. All statistics were performed with Prism 6 statistical software (GraphPad Software). Statistical significance was set at *P* < 0.05.

## Results

### IGF-1 and IGFBP levels of *Igfbp4*^−/−l^ mice

**Figure 1:**
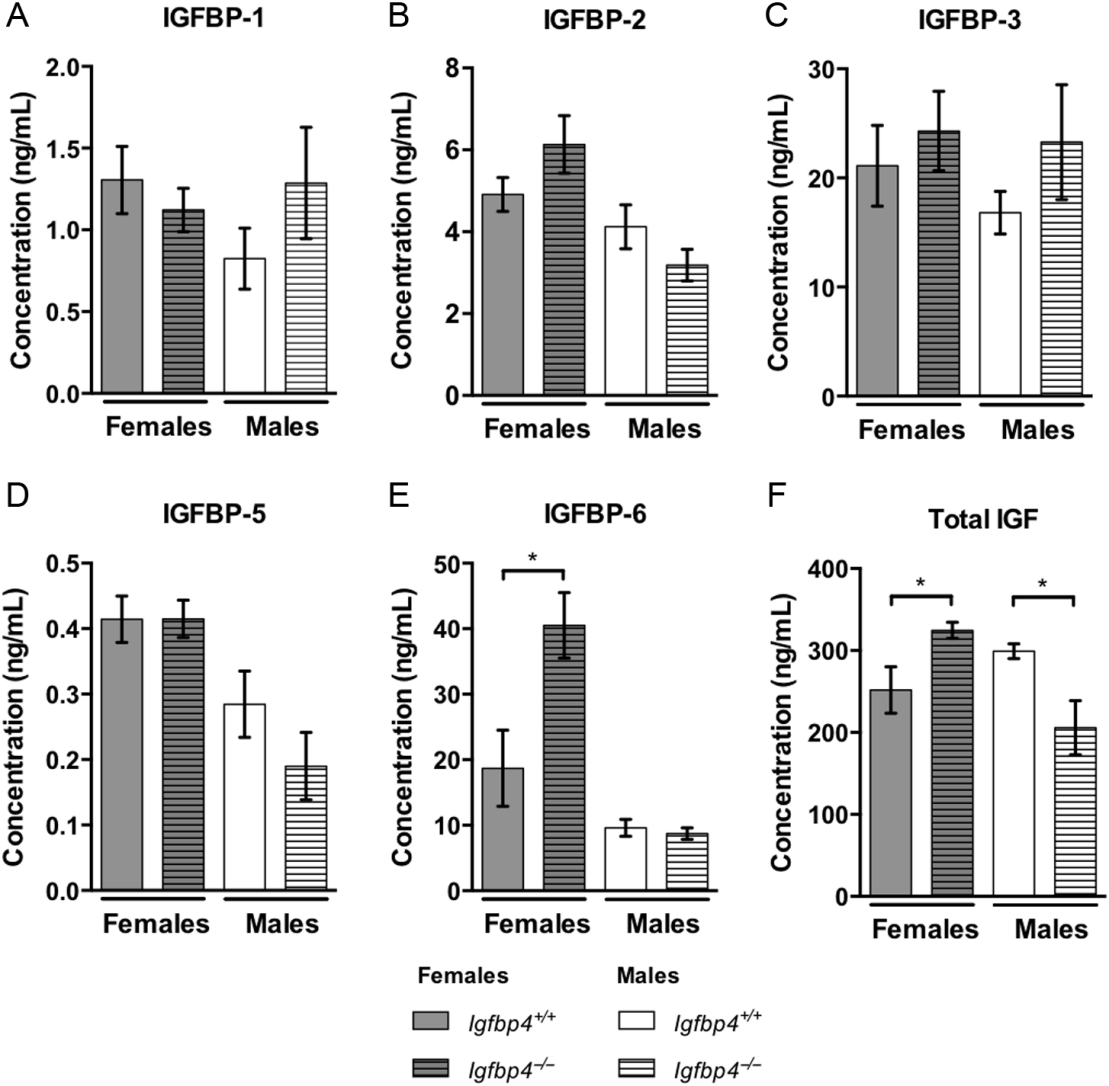
Serum proteins. (A, B, C, D and E) Circulating levels of IGFBP1, IGFBP2, IGFBP3, IGFBP5 and IGFBP6 in serum from 16-week-old mice were measured using a Milliplex magnetic bead panel. (F) Circulating levels of total (free and bound) IGF-I in serum from 16-week-old mice were measured by ELISA. (*n* = 9/genotype/gender, *t-test *P* value <0.05.)

The deletion of the exon 2 of *Igfbp4* resulted in a loss of *Igfbp4* expression in adipose tissues, liver and femur (Supplementary Fig. 1, see section on supplementary data given at the end of this article). There were no alterations in the serum levels of IGFBP-1, 2, 3 and 5 with loss of *Igfbp4* (Fig. 1A, B, C and D); however, an increase of IGFBP-6 was detected in the serum of *Igfbp4*^−/−^ females when compared to that in controls (Fig. 1E). At 16 weeks of age, total circulating IGF-1 levels were increased in *Igfbp4*^−/−^ females (324.6 ± 9.8 ng/mL vs 251.4 ± 28.3 ng/mL *P* = 0.03, Fig. 1F) but decreased in males (299.1 ± 8.8 ng/mL vs 205.8 ± 33.0 ng/mL vs 299.1 ± 8.8 ng/mL *P* = 0.01, Fig. 1F).

### Growth retardation of *Igfbp4*^−/−^ mice

**Figure 2:**
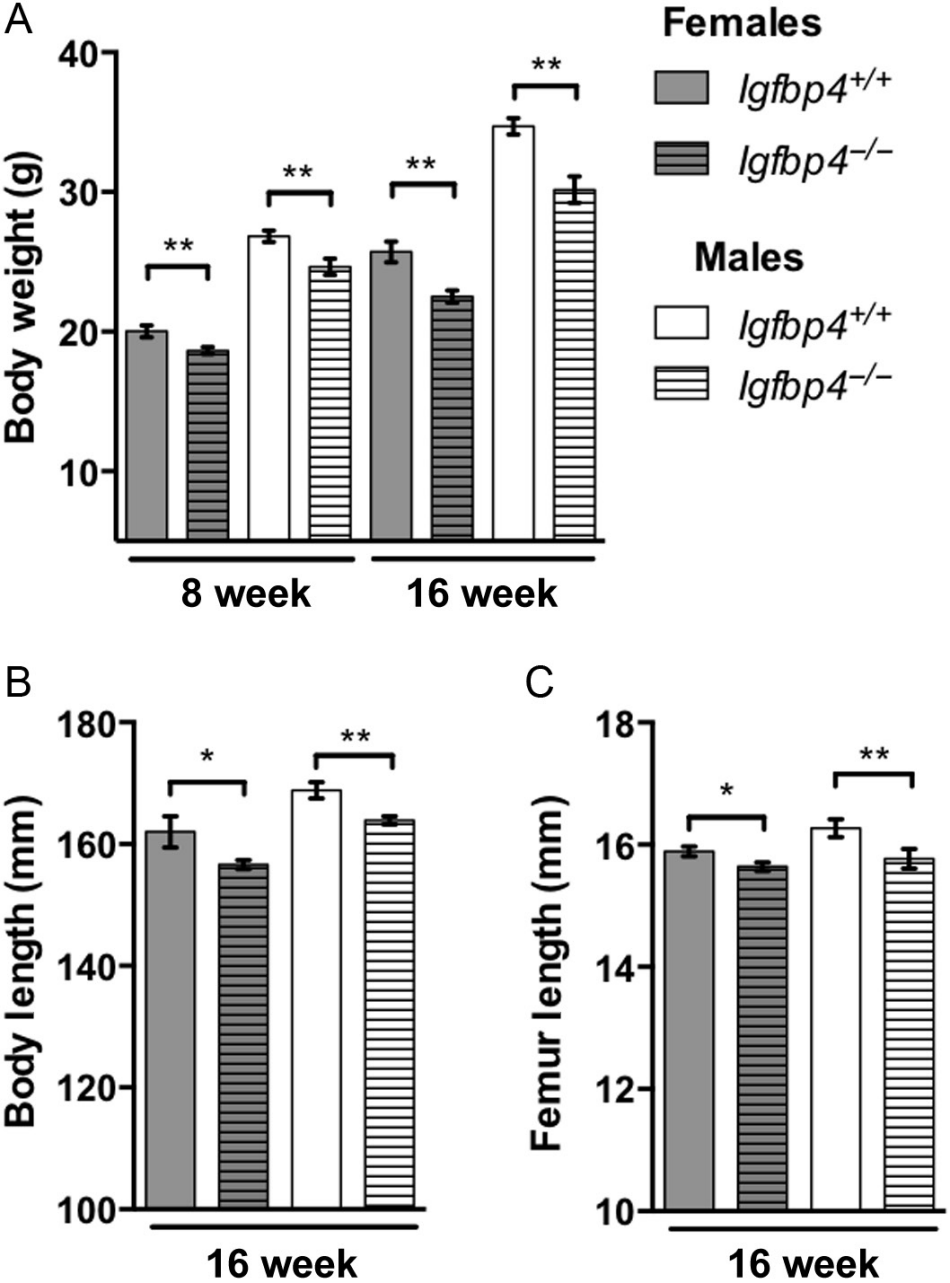
Growth retardation of *Igfbp4*^−/−^ mice. (A) Animals were weighed at 8 and 16 weeks of age. (B) Full body (tip of the nose to anus) length was measured on killed 16-week-old animals. (C) Lengths of the femurs were measured after isolation and fixation of the femurs in 10% neutral buffered formalin. (*n* = 8/genotype/gender, *t-test *P* value <0.05, **t-test *P* value<0.01.)

**Figure 3:**
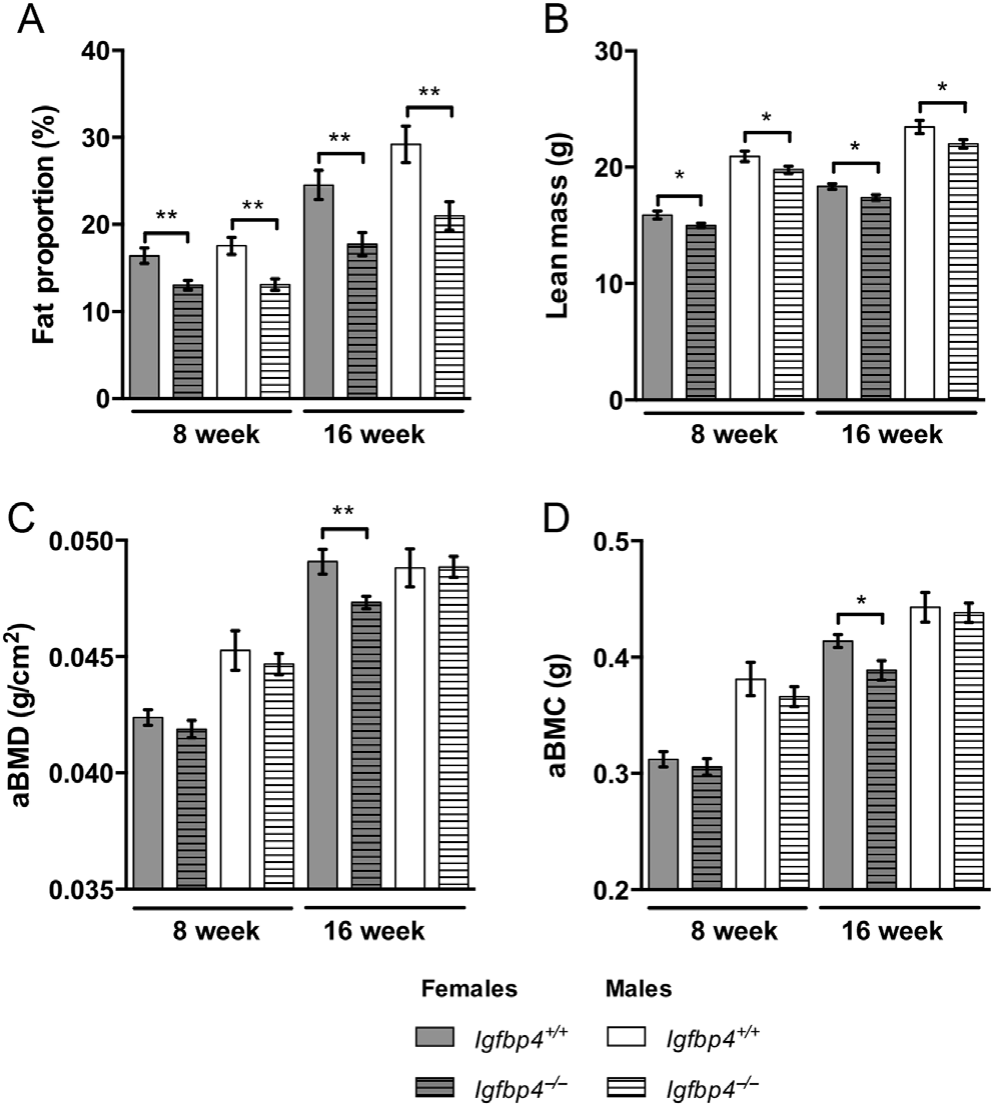
Body composition of *Igfbp4*^−/−^ mice. DXA analysis of *Igfpb4*^−/−^ mice measured at 8 and 16 weeks of age. (A) Fat proportion is calculated as the percent of fat within the entire body of the animal excluding the head. (B) Lean mass is estimated as the total tissue mass without the fat mass. (C and D) Bone parameters were measured in the entire body excluding the head. aBMC, areal bone mineral content; aBMD, areal bone mineral density. (*n* = 10/genotype/gender, *t-test *P* value <0.05, **t-test *P* value <0.01.)

*Igfbp4*^−^*^/^*^−^ mice had a significant reduction in body weight at 8 (females: 20.02 ± 0.43 g vs 18.64 ± 0.25 g, *P* = 0.008, males: 26.83 ± 0.41 g vs 24.64 ± 0.58 g, *P* = 0.008, Fig. 2A) and 16 weeks of age (females: 25.72 ± 0.74 g vs 22.51 ± 0.44 g *P* = 0.0009, males: 34.71 ± 0.58 g vs 30.17 ± 0.96 g, *P* = 0.001, Fig. 2A). *Igfbp4*^−/−^ mice were smaller with shorter body length (females: 162.0 ± 2.54 mm vs 156.6 ± 0.74 mm *P* = 0.02, males: 168.8 ± 1.35 mm vs 163.9 ± 0.68 mm, *P* = 0.003) and femur length (females: 15.9 ± 0.08 mm vs 15.6 ± 0.07 mm *P* = 0.04, males: 16.3 ± 0.14 mm vs 15.8 ± 0.16 mm, *P* = 0.03) at 16 weeks of age (Fig. 2B and C). To investigate whether the decreased growth of *Igfbp4*^−/−^ mice was affecting body compartments differentially, we used DXA to assess body composition. At 8 and 16 weeks of age, significant reductions of fat proportion and lean mass were observed in *Igfbp4*^−/−^ mice (Fig. 3A and B). There were no significant differences in aBMD or aBMC in 8-week-old animals. However, at 16 weeks of age, *Igfbp4*^−/−^ females had significant reductions in aBMD (0.049 ± 0.0005 g/cm^2^ vs 0.047 ± 0.0003 g/cm^2^, *P* = 0.006, Fig. 3C) and aBMC (0.41 ± 0.006 g vs 0.39 ± 0.008 g, *P* = 0.03, Fig. 3D) compared to littermate controls. *Igfbp4*^−/−^ males had no significant differences in these parameters at both ages (Fig. 3C and D). Taken together, our results are concordant with a previous report by Ning and coworkers in which mice with deletion of *Igfbp4* presented with postnatal growth retardation (Ning *et al*. 2008). However, in 16-week-old mice, our data suggest that females and males are affected differently at the skeletal level and this difference appears after the postnatal growth period.

### Gender-specific bone phenotype *Igfbp4*^−/−^ mice

**Figure 4:**
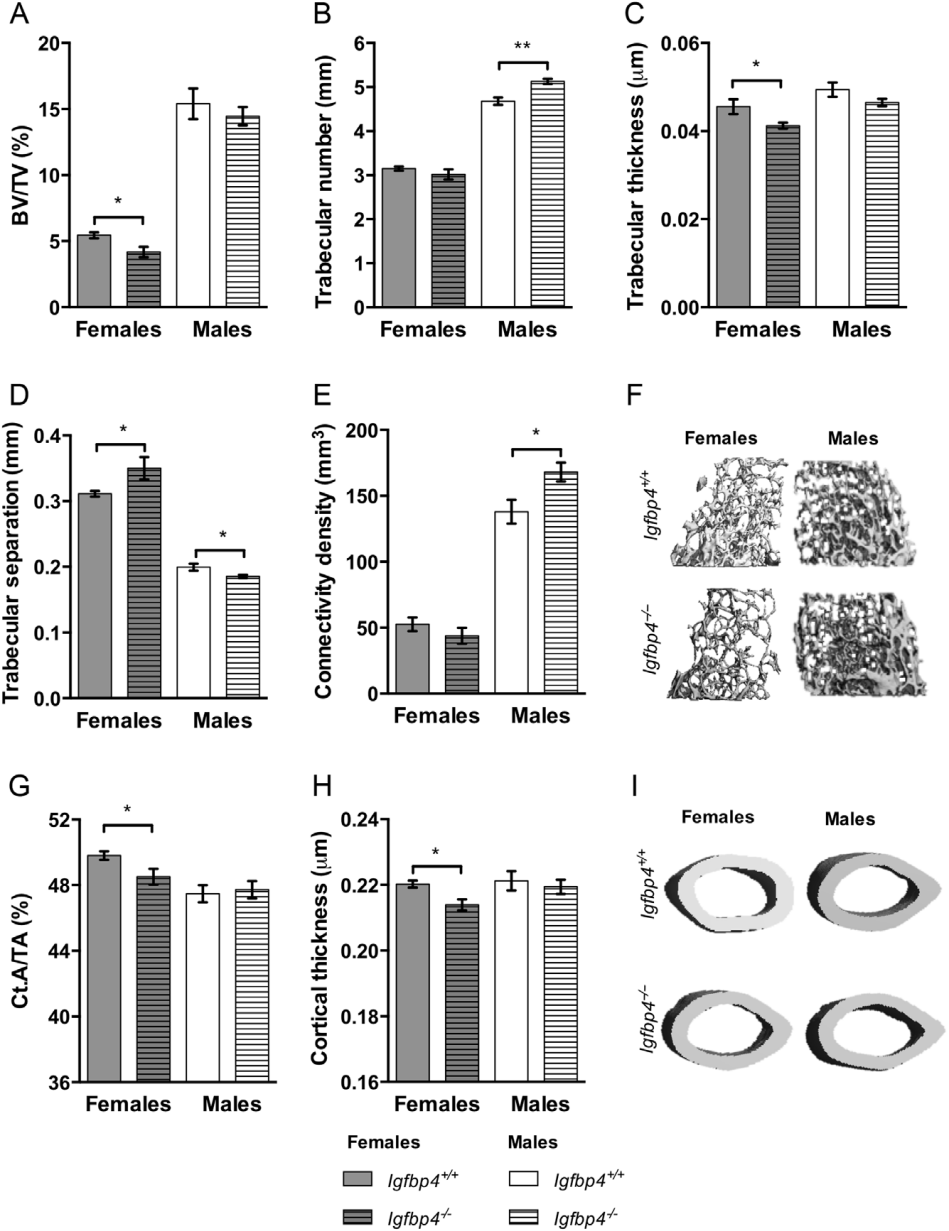
Alterations of the femoral architecture of *Igfbp4*^−/−^ mice. μCT analysis of femurs from 16-week-old animals was performed using VivaCt-40. (A, B, C, D and F) Trabecular parameters and representative pictures of trabecular bone. (G, H and I) Cortical measurements and representative pictures of cortical bone. BV/TV, bone volume/total volume; Ct.A/TA, cortical area/total area. (*n* = 8–10/genotype/gender, *t-test *P* value <0.05, **t-test *P* value <0.01.)

To investigate the microarchitecture of bone, μCT analysis was performed on femurs of 16-week-old mice. Trabecular BV/TV (5.435 ± 0.225% vs 4.170 ± 0.394%, *P* = 0.02) in the distal compartment of the femur was significantly decreased in *Igfbp4*^−/−^ females when compared to littermate controls (Fig. 4A). Trabecular thickness was also decreased in *Igfbp4*^−/−^ females (Fig. 4B), whereas trabecular separation was increased (Fig. 4D). Significant reductions were also observed in cortical area fraction (Ct.A/TA: 49.80 ± 0.26% vs 48.52 ± 0.48%, *P* = 0.03, Fig. 4G) and cortical thickness (0.2203 ± 0.001 μm vs 0.2139 ± 0.002 μm, *P* = 0.009, Fig. 4H) at the femur midshaft. Despite showing no change in trabecular BV/TV and thickness, *Igfbp4*^−/−^ males had significantly higher trabecular number (4.68 ± 0.08/mm vs 5.13 ± 0.06/mm, *P* = 0.0003, Fig. 4B) and connectivity density (138.0 ± 9.09 mm^3^ vs 168.1 ± 7.12 mm^3^, *P* = 0.02, Fig. 4E) than littermate controls. Unlike *Igfbp4*^−/−^ females, males did not show alterations of cortical bone with deletion of *Igfbp4* (Fig. 4G and H). Thus, our results indicate that there is a clear gender-specific bone phenotype observed in adult mice with loss of *Igfbp4*.

### Histomorphometry of *Igfbp4*^−/−^ mice

We performed histomorphometry on the proximal tibia to examine whether deletion of *Igfbp4* resulted in different alterations in the multicellular components of bone remodeling in males and females. Histomorphometry analysis of *Igfbp4*^−/−^ females and littermate controls were partially concordant with the μCT results (Table 1). Indeed, bone formation parameters (MS/BS, MAR, BFR and OS/BS) tended to be lower in *Igfbp4*^−/−^ females; yet, only differences in mineral apposition rate reached statistical significance. Interestingly, the number and surface of osteoblasts were modestly increased with the deletion of *Igfbp4* in females (Table 1). However, the numbers and surface of osteoclasts were also markedly higher leading to a significant increase in eroded surface in *Igfbp4*^−/−^ females (Table 1). Thus, those increases in osteoclast numbers and surfaces accompanied by the decrease of mineralization might explain the significant reduction of cortical and trabecular bone observed in *Igfbp4*^−/−^ females.

**Table 1:** Histomorphometry of *Igfbp4*^−/−^ mice.

	**Females**			**Males**		
	*Igfbp4*+/+	*Igfbp4*^−/−^	*t*-Test	*Igfbp4*+/+	*Igfbp4*^−/−^	*t*-Test
MS/BS (%)	38.7 ± 1.77	36.21 ± 1.31	0.076	**41.16 ± 2.74**	**28.46 ± 3.59**	**0.049**
MAR (µm/day)	**1.82 ± 0.02**	**1.63 ± 0.07**	**0.037**	1.04 ± 0.06	0.93 ± 0.04	0.215
BFR (µm^3^/µm^2^/day)	0.68 ± 0.06	0.59 ± 0.04	0.216	0.41 ± 0.04	0.30 ± 0.03	0.062
Ob.S/B.Pm	12.77 ± 1.21	16.10 ± 2.10	0.207	2.96 ± 0.81	1.68 ± 0.35	0.139
N.Ob/B.Pm	8.72 ± 0.83	10.92 ± 1.31	0.173	2.24 ± 0.64	1.66 ± 0.31	0.405
OS/BS µ	9.21 ± 1.38	7.81 ± 1.42	0.485	**11.03 ± 1.31**	**4.28 ± 1.09**	**0.003**
O.Th	2.58 ± 0.12	2.59 ± 0.12	0.158	2.36 ± 0.19	2.11 ± 0.13	0.308
Oc.S/BS	**12.3 ± 0.79**	**14.97 ± 0.60**	**0.022**	6.99 ± 0.46	6.69 ± 0.92	0.782
N.Oc/B.Pm	**4.24 ± 0.18**	**5.05 ± 0.21**	**0.016**	2.95 ± 0.17	2.77 ± 0.31	0.624
ES/BS	**3.96 ± 0.35**	**6.38 ± 0.99**	**0.048**	1.23 ± 0.37	1.90 ± 0.45	0.282
N.Ad/T.Ar	42.73 ± 8.16	50.12 ± 4.19	0.482	5.13 ± 2.29	5.79 ± 2.94	0864

Static and dynamic histomorphometry analysis of proximal tibia labeled with calcein and demeclocycline from *Igfbp4*^−/−^ mice compared to littermate controls at 16 weeks of age. Bold values represent measurements for which the difference from littermate gender-matched controls reach statistical significance.

BFR/BS, bone formation rate/bone surface; ES/BS, erosion surface/bone surface; MAR, mineral aposition rate; MS/BS, mineralized surface/bone surface; N.Ad/T.Ar, number of adipocyte/total area; N.Ob/B.Pm, number of osteoblast/bone perimeter; N.Oc/B.Pm, number of osteoclast/bone perimeter; Ob.S/B.Pm, osteoblast surface/bone perimeter; Oc.S/BS, osteoclast surface/bone surface; OS/BS, osteoid surface/bone surface; O.Th, osteoid thickness. (*n* = 5–6/genotype/gender, *t*-test *P* values were considered statistically different when *P* value <0.05.)

There was a decrease in osteoid surface per bone surface and mineralization surface in 16-week-old *Igfbp4*^−/−^ males (Table 1). However, there were no significant differences in the number of osteoblasts and osteoclasts compared to those in littermate controls (Table 1). Taken together, our data show that loss of *Igfbp4* affects bones of female and male mice differentially with distinct alterations in cells of the bone-forming unit.

### Ovariectomy-induced bone loss is blunted in *Igfbp4*^−/−^ mice

**Figure 5:**
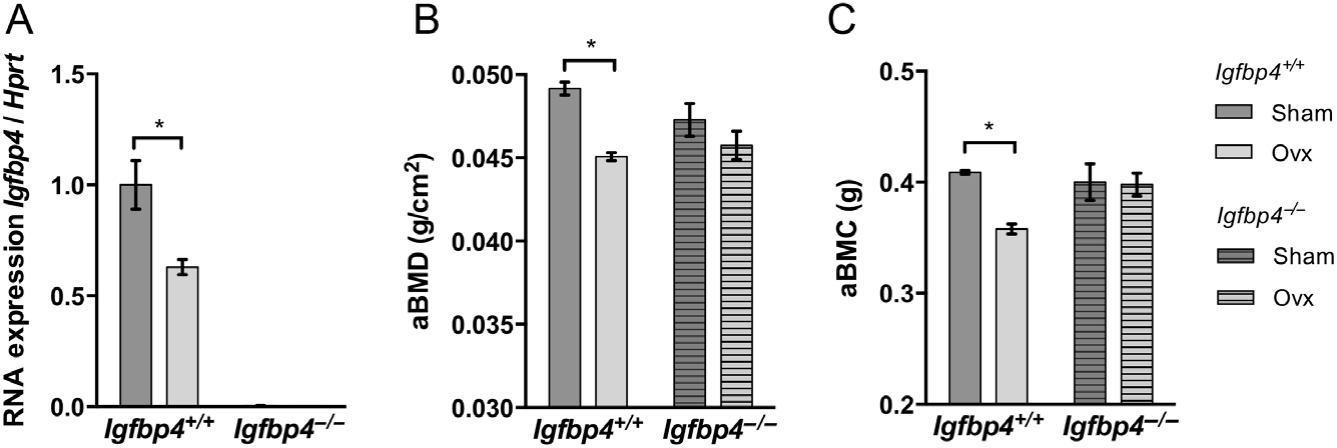
Ovariectomy-induced bone loss. Female *Igfbp4^+/+^* and *Igfbp4*^−^*^/^*^−^ mice ovariectomized at 8 weeks of age RNA and killed at 16 weeks of age. (A) RNA was isolated from femurs and subjected to quantitative PCR to observe *Igfbp4* gene expression. (B and C) Animals were subjected to DXA at 16 weeks of age to observe bone parameters. aBMC, areal bone mineral content; aBMD, areal bone mineral density. (*n* = 5–8/surgery group/genotype, *two-way ANOVA *P* value <0.05.)

Previous studies have reported that estrogen could regulate *Igfbp4* transcription (Kassem *et al*. 1996, Qin *et al*. 1999, Wang *et al*. 2004, Mita *et al*. 2007). Moreover, estrogen is known to regulate bone resorption and osteoclastogenesis (Kameda *et al*. 1997, Riggs 2000, Kondoh *et al*. 2014). Thus, we postulated that estrogen could regulate *Igfbp4* expression within the bone, which could explain that gender-specific phenotype. To test our hypothesis, we ovariectomized 8-week-old *Igfbp4*^−^*^/^*^−^ females and littermate controls to examine bone loss observed during estrogen depletion. *Igfbp4* mRNA level was significantly decreased in femurs of *Igfbp4^+/+^* mice after 8 weeks of ovariectomy (Fig. 5A). *Igfbp4^+/+^* ovariectomized females had a significant loss of aBMD and aBMC compared to those in sham-operated animals (aBMD: 0.049 ± 0.0004 g/cm^2^ vs 0.045 ± 0.002 g/cm^2^, *P* = 0.006, aBMC: 0.41 ± 0.002 g vs 0.36 ± 0.005 g, *P* = 0.01, Fig. 5B and C). However, total aBMD and aBMC were not significantly different between *Igfbp4*^−^*^/^*^−^ ovariectomized females and sham-operated controls (Fig. 5B and C).

**Figure 6:**
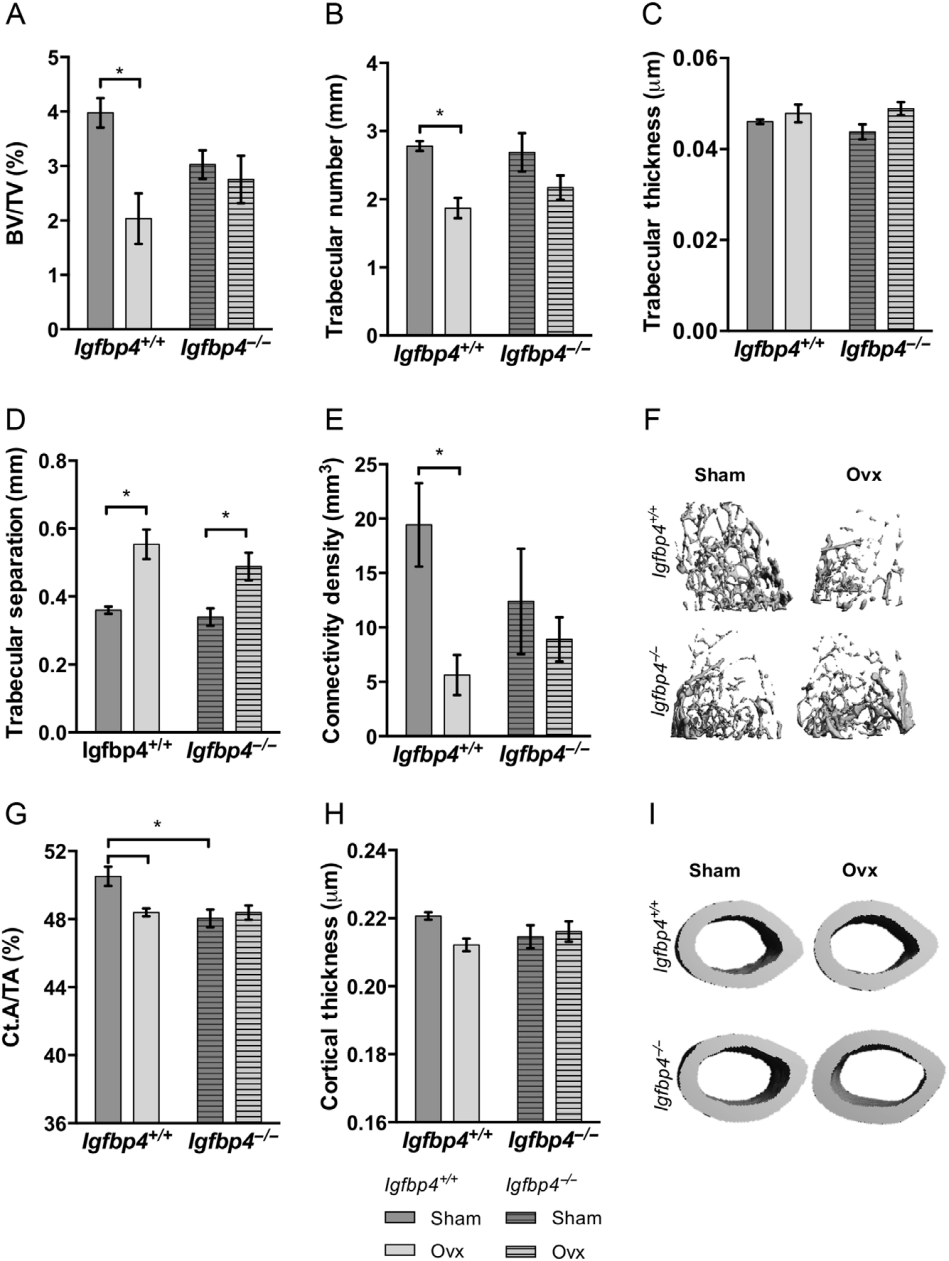
Femoral architecture upon loss of estrogen. Fixed femurs isolated from 16-week-old ovariectomized or sham-operated mice were analyzed by μCT to examine trabecular (A, B, C, D, E and F) and cortical (G, H and I) microarchitecture. BV/TV, bone volume/total volume; Ct.A/TA, cortical area/total area; OVX, ovariectomized cohort. (*n* = 5–8/surgery group/genotype, *two-way ANOVA *P* value <0.05.)

μCT analysis revealed markedly decreased trabecular bone volume in *Igfbp4^+/+^* ovariectomized mice (Fig. 6A, B, C, D, E and F). Albeit for trabecular separation, which was increased in both *Igfbp4*^−^*^/^*^−^** and *Igfbp4^+/+^* mice upon ovariectomy (Fig. 6D), no changes were observed between sham-operated and ovariectomized *Igfbp4*^−^*^/^*^−^** mice. Similarly, cortical area fraction (Fig. 6G) and cortical thickness (Fig. 6H) were reduced upon ovariectomy in *Igfbp4^+/+^* mice but not in *Igfbp4*^−^*^/^*^−^** mice. These data indicate that *Igfbp4*^−^*^/^*^−^ females are partially protected against trabecular bone loss caused by estrogen deficiency.

### Primary marrow cell cultures

**Figure 7:**
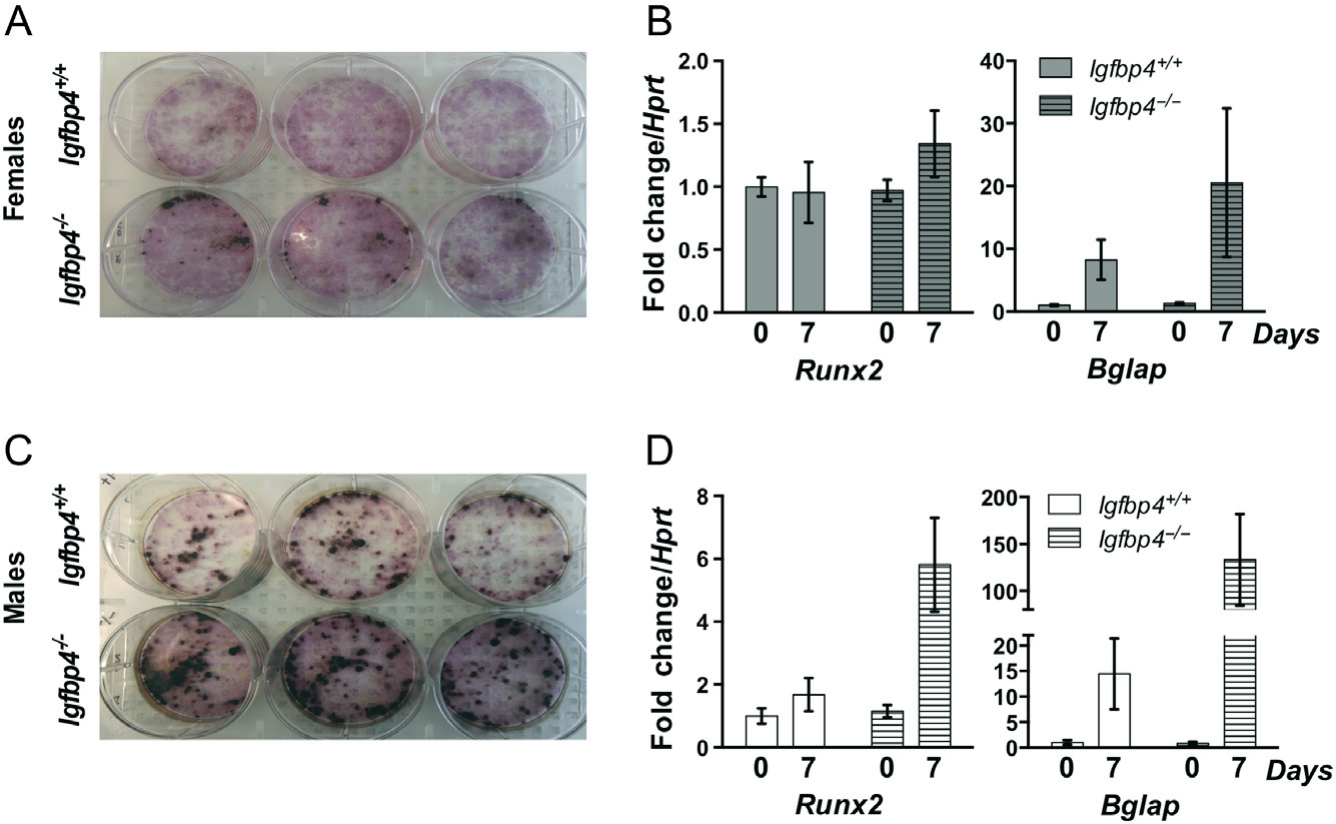
Primary cultures of BMSCs. BMSCs differentiated into osteoblasts were isolated from long bones from 8-week-old *Igfbp4*^−/−^ and *Igfbp4^+/+^* females (A and B) and males (C and D). Osteoblasts were stained for alkaline phosphatase in pink and Von Kossa was performed to show mineralization in black (A and C). RNA was isolated from culture wells in order to examine gene expression of *Runx2* and *Bglap* (B and D). (*n* of mice = 3/gender/genotype/experiments, *n* of wells = 3/gender/genotype.)

**Figure 8:**
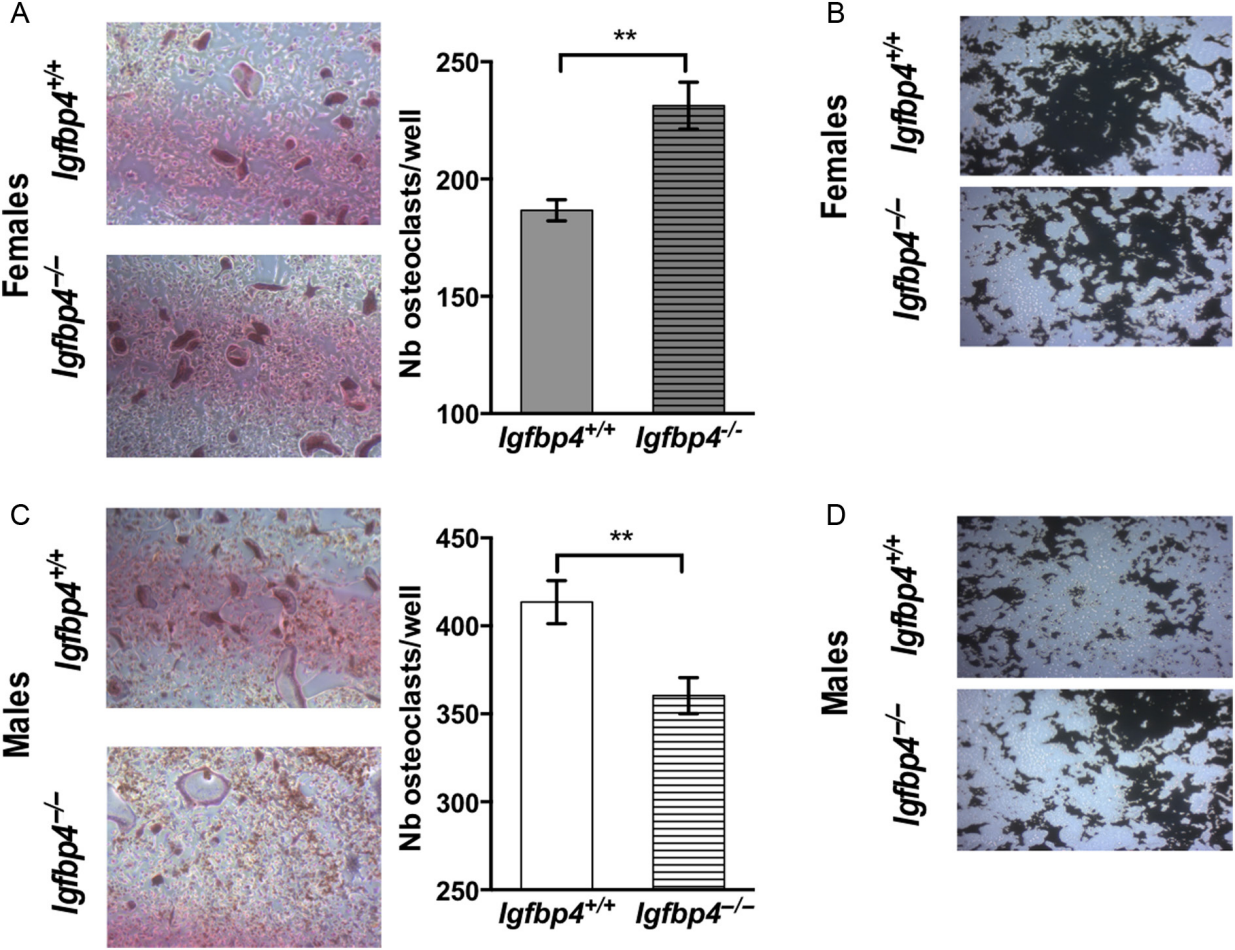
Primary cultures of HSCs. HSCs differentiated into osteoclasts were isolated from long bones from 8-week-old *Igfbp4*^−/−^ and *Igfbp4^+/+^* females and males. (A and C) HSCs were differentiated into osteoclasts for 14 days in 24-well plates. Osteoclasts were counted in each culture well as large TRAP-positive purple cells. (B and D) Representative pictures of resorbed area (white) by osteoclasts cultures on a mineral surface labelled by Von Kossa (black). (*n* of mice = 3/gender/genotype/experiments, *n* of wells = 6–7/gender/genotype, **t-test *P* value <0.01.)

To resolve whether the gender-specific alterations of the bone-forming unit caused by loss of *Igfbp4* could be recapitulated *in vitro*, we isolated BMSCs and HSCs from *Igfbp4*^−/−^ and *Igfbp4^+/+^* mice for evaluation of osteoblastogenesis and osteoclastogenesis *in vitro*. Consistent with the increase in osteoblast and osteoclast numbers noted by histomorphometry, there was an increase in osteoblastogenesis (Fig. 7A and B) and osteoclastogenesis (Fig. 8A) in cells isolated from *Igfbp4*^−/−^ females compared to those from *Igfbp4^+/+^*. Also, female *Igfbp4*^−/−^ osteoclasts appeared to have a higher resorbing activity than controls (Fig. 8B). Although male *Igfbp4*^−/−^ BMSCs showed greater osteoblastogenesis (Fig. 7C and D), there was a reduction of osteoclastogenesis and osteoclastic resorption compared to that in controls (Fig. 8C and D). Taken together, the *in vitro* data support the hypothesis that loss of *Igfbp4* affects the differentiation of progenitor cells and the functions of osteoblasts and osteoclasts.

### Femoral gene expression and circulating protein levels in *Igfbp4*^−/−^ mice

**Figure 9:**
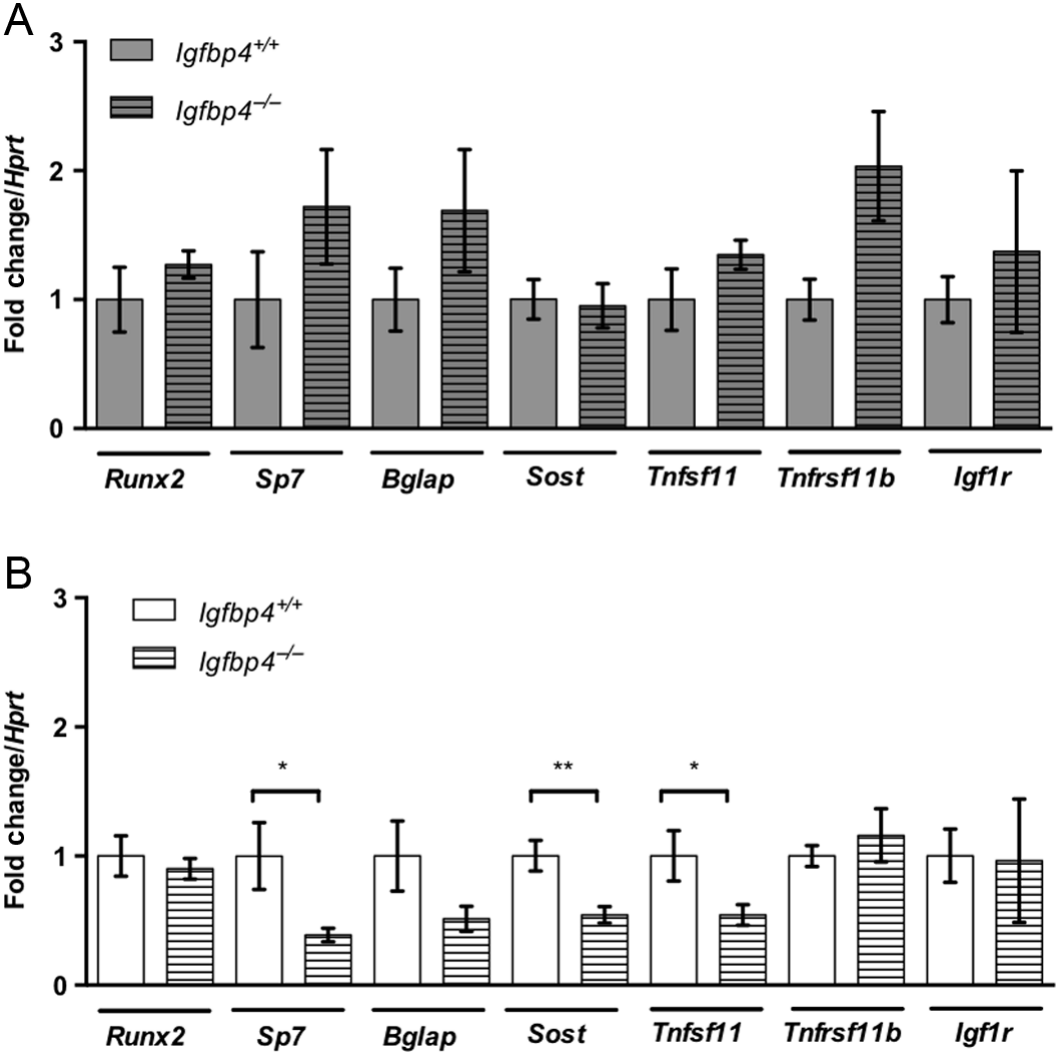
Gene expression in femurs from *Igfpb4*^−/−^ mice. cDNA samples obtained from RNA from whole femurs of 16-week-old female (A) and male (B) mice were subjected to quantitative PCR to investigate the expression bone-related genes. (*n* = 6–8/genotype/gender, *t-test *P* value <0.05, **t-test *P* value <0.01.)

Because loss of *Igfbp4* affected males and females differently, we hypothesized that molecular mechanisms regulating bone were also altered in a gender-specific fashion. To address this question, we first examined the expression of bone-related genes: *Runx2*, *Sp7*, *Bglap*, *Sost*, *Tnfsf11*, *Tnfrsf11b* and *Igf1r* in the femurs of 16-week-old *Igfbp4*^−/−^ and *Igfbp4^+/+^* mice. Although no significant changes were detected in *Igfbp4*^−^*^/^*^−^ females (Fig. 9A), *Igfbp4*^−^*^/^*^−^ males showed decreased expressions of *Sp7* and *Sost* and *Tnfs11* when compared to *Igfbp4^+/+^* males (Fig. 9B). The downregulation of *Sp7*, coding for Osterix, could be consistent with the lower mineralization revealed by histomorphometry (Table 1).

**Figure 10:**
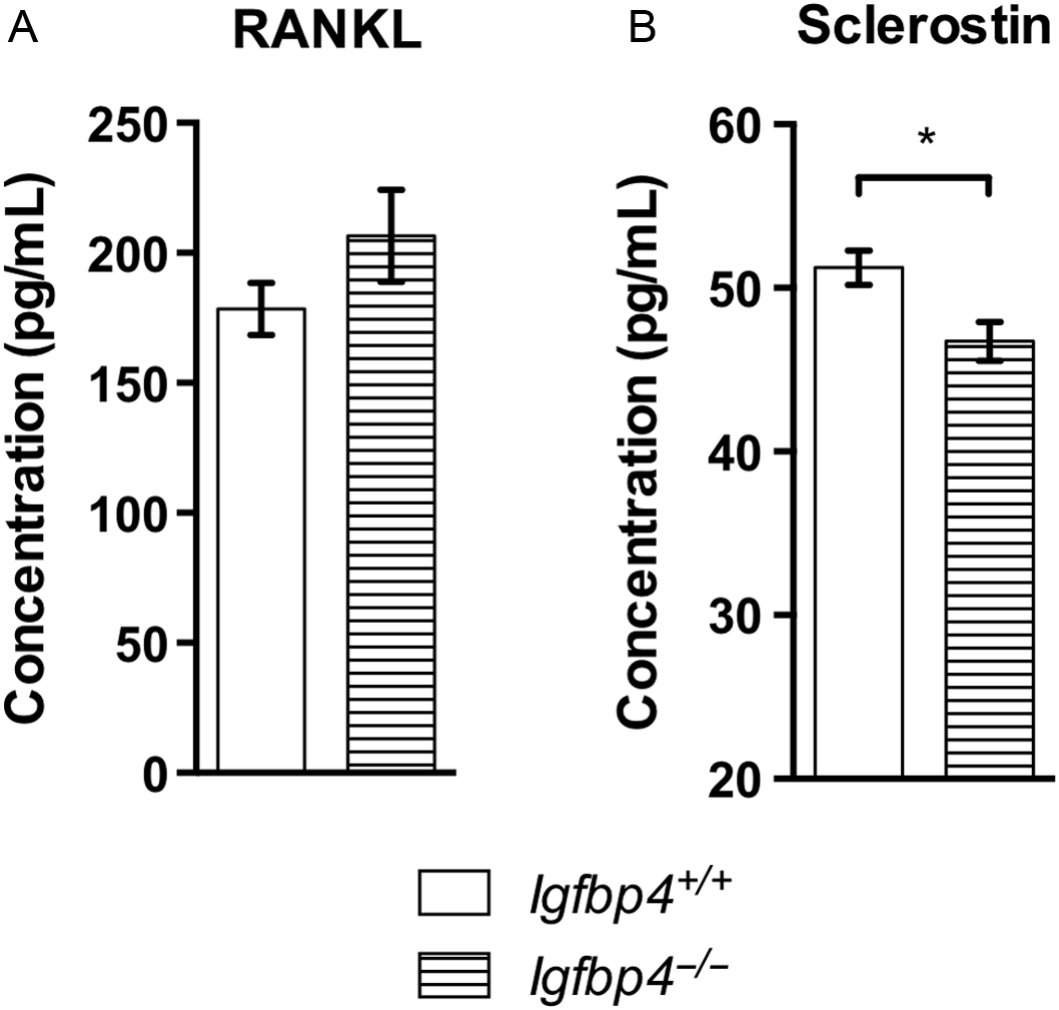
Circulating levels of RANKL and Sclerostin in *Igfpb4*^−/−^ mice. RANKL (A) and Sclerostin (B) levels were measured by ELISA on sera samples from 16-week*-*old *Igfpb4*^−/−^ males and littermate controls. (*n* = 10/gender/genotype/time point, *t-test *P* value <0.05, **t-test *P* value <0.01.)

Next, we tested whether the reductions of *Tnfs11* and *Sost*, coding for RANKL and Sclerostin, respectively, were reflected at the circulating level. Measurement of RANKL in the serum of male mice showed no differences between *Igfbp4*^−^*^/^*^−^ mice and controls (Fig. 10A). However, we observed a mild but significant decrease of circulating Sclerostin at 16 weeks of age in *Igfbp4*^−^*^/^*^−^ males (51.24 ± 1.06 pg/mL vs 46.74 ± 1.17 pg/mL, *P* = 0.01, Fig. 10B). Taken together, our results indicate that IGFBP-4 is important for normal growth in adult mice. IGFBP-4 appears to be important for the development and maintenance of bone by locally affecting osteoblast and osteoclast function in a gender-specific manner.

## Discussion

In this study, we hypothesized that IGFBP-4 might play an important role in sequestering IGF-I in the adult skeleton and therefore act distinctly *in vivo* vs *in vitro*. Instead, we found evidence that IGFBP-4 can act in both a cell autonomous and cell non-autonomous manner and sex specifically to influence the adult skeleton. This is in sharp contrast to the earliest characterization of the IGF-binding proteins, particularly, IGFBP-4 that has been considered within the framework of an inhibitory protein that blocked IGF-1 and IGF-2 actions in various cell systems (Culouscou & Shoyab 1991, Schiltz *et al*. 1993, Gustafsson *et al*. 1999). Previous attempts at studying the *in vivo* actions of IGFBP-4 were limited to characterizing murine embryonic and postnatal mechanisms of IGFBP-4 without specifically looking at sexual dimorphism (Ning *et al*. 2006, 2008). In contrast, we examined pubertal and adult skeletal maintenance in C57BL/6J mice by phenotyping mice with a germline deletion of the *Igfbp4* gene. In this study, we show that IGFBP-4 is required for the sustained growth of adult female and male mice. We found that loss of *Igfbp4* resulted in growth retardation marked by decreased fat proportion, lean mass, body length and body weight. An elegant study performed by Ning and coworkers previously reported the growth retardation caused by deletion of Igfbp4 starts during embryonic life and persists up to 14 weeks of age (Ning *et al*. 2008). Interestingly, although no alterations of the bone mass were detected by DXA at 8 weeks of age, we noted that 16-week-old *Igfbp4*^−^*^/^*^−^ females had significant decreases in aBMD and aBMC when compared to those in littermate controls (Fig. 3C and D). This suggests that IGFBP-4 plays a role in regulating bone acquisition during the post-pubertal growth period (i.e. between 8 and 16 weeks of age) in mice.

Interestingly, the growth phenotype of both males and females was not concordant with the circulating total IGF-1 levels (Fig. 1F). Indeed, although both genders suffered from growth retardation, only *Igfbp4*^−^*^/^*^−^ males had a significant reduction in serum IGF-1 levels, whereas *Igfbp4*^−^*^/^*^−^ females showed increased circulating levels of IGF-1. Thus, the growth retardation phenotype may not be caused by a systemic alteration of IGF-1 in female mice. However, serum levels of IGFBP-6 were also markedly increased in *Igfbp4*^−^*^/^*^−^ females (Fig. 1E). IGFBP-6 has a higher affinity for IGF-2 than IGF-1 (Gustafsson *et al*. 1999, Konermann *et al*. 2013, Raykha *et al*. 2013). IGF-2 is principally involved in the regulation of placental and fetal growth in mice and its expression is highest in the brain of adult mice (Nielsen 1992, Constancia *et al*. 2002, Ferron *et al*. 2015). Thus, we suspect that the increase observed in circulating levels of IGFBP-6 might not have a significant impact on adult growth of *Igfbp4*^−^*^/^*^−^ females. Furthermore, it has been shown that IGFBP-6 could inhibit osteoblast differentiation (Strohbach *et al*. 2008). Yet, *Igfbp4*^−^*^/^*^−^ females showed increased osteoblastogenesis by histomorphometry (Table 1) suggesting that the increased circulating IGFBP-6 levels cannot explain the skeletal phenotype of *Igfbp4*^−^*^/^*^−^ females. Instead, we propose that tissue-specific regulation of IGF-1 might be causing the skeletal phenotype.

To our knowledge, this is the first study to systematically examine the bones of adult *Igfbp4*^−^*^/^*^−^ mice. Here, we show that the deletion of *Igfbp4* resulted in a sex-specific modification of bone microarchitecture with reduced cortical and cancellous bone in females but a mild increase in trabeculae in males (Fig. 4). Histomorphometry of the tibia revealed that *Igfbp4*^−^*^/^*^−^ females had more osteoclasts resulting in increased eroded surface/bone surface but decreased bone formation suggesting a significant uncoupling in the bone remodeling unit (Table 1). In contrast, *Igfbp4*^−^*^/^*^−^ males had more trabeculae but the struts were thinner and there were no alterations in bone volume fraction (Fig. 4).

Based on our observations about the sex differences, we hypothesized that the bone loss observed in female *Igfbp4*^−^*^/^*^−^ mice might be related to an alteration in estrogen regulation of IGFBP-4. In fact, it has been shown that estrogen induces *Igfbp4* gene transcription in osteoblastic and breast cancer cells (Kassem *et al*. 1996, Qin *et al*. 1999). Therefore, deletion of *Igfbp4* in females could recapitulate an estrogen deficiency state. This is supported by the increase in bone turnover observed in *Igfbp4*^−^*^/^*^−^ females (Table 1). Similarly, ovariectomized *Igfbp4*^−^*^/^*^−^ mice did not lose trabecular bone as their littermate controls did (Figs 5 and 6). This could be due to the fact that *Igfbp4*^−^*^/^*^−^ females have low bone mass independent of estrogen withdrawal and thus, the effects on the skeleton from ovariectomy are minimal compared to ovariectomy in *Igfbp4^+/+^* females. On the other hand, this may imply that loss of IGFBP4 indirectly reflects loss of estrogen signaling to bone cells. Notwithstanding, estrogen does not appear to be the only regulator of *Igfbp4* within bone or the circulation as ovariectomy does not completely obliterate *Igfbp4* expression (Fig. 5A). Taken together, our findings are consistent with other studies of the IGFBPs suggesting that estrogen can target IGF-binding proteins as one means of regulating IGF signaling in bone cells.

The cell autonomous actions of IGFBP-4 are potentially very important. Histomorphometric studies pointed to significant skeletal effects from global loss of *Igfbp4.* Thus, we examined the process of osteoclastogenesis and osteoblastogenesis *in vitro* using primary cells from controls and mutants. Our *in vitro* cultures of HSCs recapitulated the phenotype we detected by histomorphometry of greater osteoclastogenesis and a larger eroded surface in *Igfbp4*^−^*^/^*^−^ females (Fig. 8A and B). On the other hand, bone marrow stromal cell cultures revealed an enhanced osteogenic capacity compared to wild-type controls (Fig. 7). Bone marrow stromal cells isolated from *Igfbp4*^−/−^ male mice also showed increased osteoblastogenesis and mineralization but decreased osteoclastogenesis. The decrease in osteoclasts might explain why *Igfbp4*^−/−^ males have increased trabecular number. However, it is likely that with higher rates of osteogenesis, *Igfbp4*^−/−^ males could maintain cortical and trabecular bone volumes similar to littermate controls.

Despite the increase in mineralization seen *in vitro* in cells from *Igfbp4*^−/−^ males (Fig. 7C and D), histomorphometry and gene expression analysis revealed decreased mineralization and expression of *Sp7* in femurs of *Igfbp4*^−/−^ males (Fig. 9B and Table 1). It is likely that certain aspects of the regulatory mechanism cannot be recapitulated *in vitro* in a cell autonomous fashion. In support of this hypothesis, *Sost* femoral expression (Fig. 9B) and circulating levels of Sclerostin were both decreased in 16-week-old *Igfbp4*^−/−^ males (Fig. 10B) suggesting a more systemic alteration of bone regulation in *Igfbp4*^−^*^/^*^−^ mice. Indeed, Sclerostin is an inhibitor of Wnt signaling and *Sost*-knockout mice have higher bone mass (Li *et al*. 2008). Although this decrease in Sclerostin might contribute to the increase in trabecular number revealed by μCT and histomorphometry, it does not explain why bone volume is not affected in 16-week-old *Igfbp4*^−^*^/^*^−^ males.

In summary, our study shows that *Igfbp4* is required for normal adult growth of bone and lean mass in mice. Our data also revealed that *Igfbp4* was particularly important during the period of peak bone mass acquisition, occurring between 8 and 16 weeks of age in mice. However, there is a clear sex specificity with global deletion of IGFBP-4. *Igfbp4*^−^*^/^*^−^ females do not gain bone during peak bone mass acquisition, but *Igfbp4*^−^*^/^*^−^ males are able to acquire sufficient bone mass. Loss of *Igfbp4* was sufficient to create a cell autonomous phenotype observed *in vitro* with increased osteoblastogenesis in *Igfbp4*^−^*^/^*^−^ cells. A gender dimorphism was also observed *in vitro* with osteoclastic activity being increased in cells from *Igfbp4*^−^*^/^*^−^ female mice but decreased in *Igfbp4*^−^*^/^*^−^ males. Thus, we propose that IGFBP-4 has systemic as well as tissue- and sex-specific actions on the regulation of osteoblastogenesis and osteoclastogenesis. Those actions of IGFBP-4 are most likely conserved in humans. Indeed, IGFBP-4 circulating levels were elevated in older women with hip and spine fractures (Rosen *et al*. 1992). IGFBP-4 serum levels were also correlated with bone mineral density in growth hormone-deficient adults (Thoren *et al*. 1998). Furthermore, a locus 500 bp upstream of human *Igfbp4* was found to be regulatory of height in a GWAS (Berndt *et al*. 2013). Thus, IGFBP-4 in humans as in mice appears to be important for growth but our study shows clear sexual dimorphism in mice.

Previous reports showed that overexpression of IGFBP-4 in osteoblasts decreased bone formation and femoral length (Zhang *et al*. 2003), whereas systemic injections of IGFBP-4 resulted in increased bone formation (Miyakoshi *et al*. 2001). Those studies concluded that IGFBP-4 is necessary to sequester IGF-I in the bone environment and increased its bioavailability. Our results are concordant with this proposed mechanism where IGFBP-4 is required for adult bone growth with sex specificity. One possible mechanism for the sex differences relate to the role estrogen might play in regulating IGFBP-4 expression in bone. Our study reinforces the importance of studying both sexes as well as different ages when studying skeletal phenotypes from global or conditional deletion of genes in the IGF regulatory system. Our findings also support the long-standing tenet that the IGFBPs work in a cell- and context-specific manner such that assignment of their functional significance is complex.

## Supplementary Material

Supporting Figure 1

## Declaration of interest

The authors declare that there is no conflict of interest that could be perceived as prejudicing the impartiality of the research reported.

## Funding

This work was supported by the National Institutes of Health (R01 AR061164-01A1).
