# Supplementary figures and images for: IGFBP-4 regulates adult skeletal growth in a sex-specific manner
Source: J Endocrinol. 2017 Feb 9;233(1):131–44. doi: 10.1530/JOE-16-0673 (PMC5425953; doi:10.1530/JOE-16-0673)

Femur

iWAT

gWAT

Liver

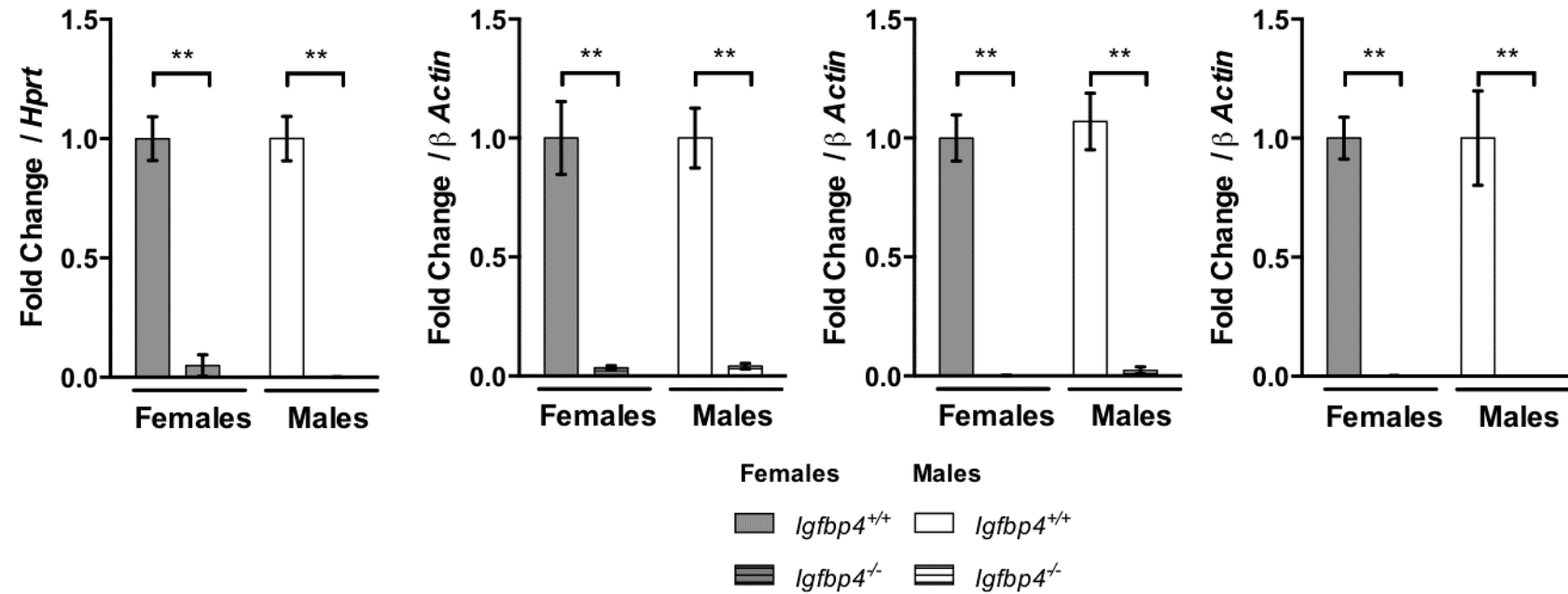

Supplement: Supporting Figure 1 [file joe-233-131-s001.pdf]
